# *Notes from the Field:* Harmful Algal Bloom Affecting Private Drinking Water Intakes — Clear Lake, California, June–November 2021

**DOI:** 10.15585/mmwr.mm7141a3

**Published:** 2022-10-14

**Authors:** Gina M. Solomon, Beckye Stanton, Sarah Ryan, Amy Little, Catherine Carpenter, Susan Paulukonis

**Affiliations:** ^1^Public Health Institute, Oakland, California; ^2^Division of Occupational and Environmental Medicine, University of California San Francisco, San Francisco, California; ^3^Office of Environmental Health Hazard Assessment, California Environmental Protection Agency; ^4^Big Valley Rancheria Environmental Protection Agency, Lakeport, California; ^5^Division of Drinking Water, Water Resources Control Board, California Environmental Protection Department; ^6^Tracking California, Public Health Institute, Oakland, California.

During June–November 2021, multiple cyanobacteria harmful algal blooms (HABs) occurred in Clear Lake, the largest freshwater body of water located entirely within the state of California. During this period, measured chemical concentrations of microcystins (a class of cyanotoxins produced by cyanobacteria) in the water of the Lower and Oaks arms of Clear Lake were persistently above the California recreational “Danger” health advisory level of 20 *μ*g/L (*1*). A maximum microcystin detection of 160,378 *μ*g/L was measured at a beach in the city of Clearlake in September 2021 (*2*). Microcystins are potent hepatotoxins and can cause gastroenteritis, dermatitis, and allergic reactions (*3*). The 17 local public drinking water systems with source lake water intakes in Clear Lake monitored and managed their systems with frequent testing, adjustment of treatment, and other measures to ensure that microcystins in finished drinking water did not exceed the Environmental Protection Agency (EPA) drinking water health advisory of 0.3 *μ*g/L (*4*). However, an unknown number of homes around Clear Lake relied on private water systems with lake water intakes or wells, and the quality of the private drinking water at those homes was unknown.

To address this uncertainty, the California Water: Assessment of Toxins for Community Health (Cal-WATCH) project collected and analyzed tap water samples for cyanotoxins from households with private lake water intakes and private wells located ≤50 ft (15.2 m) of the lake. To identify potentially eligible homes, parcel maps of lakefront land were overlaid with built structure and water system boundary maps of all 17 local public drinking water systems. Staff members from the Big Valley Rancheria Environmental Protection Department visually inspected the shoreline by boat to identify potential lake water intakes. A total of 493 parcels with structures that were not within the boundary of any public water system or that had a visible lake water intake were identified. Outreach to homeowners included postcards, door hangers, and knocking on doors. Broader community outreach was also conducted via social media and local earned media. This study was reviewed and approved by the Public Health Institute Institutional Review Board.*

Forty-six eligible homeowners enrolled in the study, and study staff members collected questionnaires and at least one water sample from each home during June to November 2021. Microcystins were not detected in the tap water of any homes with near-shore wells; however, several water samples had cyanobacteria visible on microscopy, potentially indicating lake water intrusion. Six out of the 15 well owners did not have filtration systems. In contrast, microcystins were detected in the tap water of 22 of the 31 homes with lake water intakes (Supplementary Figure, https://stacks.cdc.gov/view/cdc/121659). In 18 of these homes, the level of microsystin concentration was at or above the EPA drinking water health advisory level of 0.3 *μ*g/L (maximum = 3.9 *μ*g/L). All 31 private lake water intakes had some type of filtration; of these, 27 also had one or more forms of prefiltration treatment, including chlorination, ultraviolet disinfection, or ozone treatment (Table). Chlorination or other treatment could lyse cyanobacteria cells, releasing cyanotoxins. Although the sampling team did not inspect the treatment systems, homeowners mentioned difficulties with setup and ongoing maintenance and testing; even well-maintained systems might have been overwhelmed by the high concentrations of microcystins in the source water.

**TABLE:** Drinking water sampling results, by private water source and treatment system — Clear Lake, California, 2021

Private water source/Treatment system	No.*	Result, no.	
		Microcystin detected^†^	Microcystin ≥0.3 *μ*g/L
**Lake water intake**			
Chlorination and filtration	20	11^§^	9
Chlorination, filtration, and ultraviolet disinfection	3	3	2
Chlorination, filtration, and ozone treatment	1	1	1
Filtration only	4	4	4
Filtration and ultraviolet disinfection	2	2	1
Filtration and ozone treatment	1	1	1
**Total**	**31**	**22**	**18**
**Well**			
Chlorination and filtration	6	0	0
Filtration and ultraviolet disinfection	1	0	0
Filtration, ultraviolet disinfection, and ozone treatment	1	0	0
Filtration only	1	0	0
None	6	0	0
**Total**	**15**	**0**	**0**

* Water was sampled multiple times at several homes. For those homes, the highest result was used.

^†^ Limit of detection for most assays was 0.1 *μ*g/L.

^§^ Does not include one value from a lake water intake system with chlorination and filtration that was listed as “detected not quantifiable.”

On September 15, 2021, based on the Cal-WATCH findings and the increasing severity of HABs in the lake, the local health officer issued an emergency “Do Not Drink” advisory for private lake water drinking systems.^†^ State, local, and tribal governments coordinated with nearby public water systems to provide free drinking water filling stations for the affected population. The local health officer lifted the advisory on November 16, 2021, when levels of microcystins in most lake water samples had declined below “Danger” advisory levels.^§^ Residents were advised to flush their systems, have them professionally inspected, and have filters changed before any household use; drinking water fill stations remained open.

The frequency of HABs in drinking water sources might increase because of climate change.^¶^ During a freshwater HAB event, households with private lake water intakes are at high risk of cyanotoxin contamination. Frequent monitoring for early detection of HABs, partnerships across agencies for public health response, emergency alerts, and provision of alternative drinking water are short-term solutions. Longer-term solutions include transitioning homes to public water systems and ongoing public education about HABs. Research into other strategies to mitigate HABs, including reduction of nutrient runoff, revegetation of shorelines, and aeration, might offer additional long-term prevention opportunities (*5*).

## Footnotes

* 45 C.F.R. part 46; 21 C.F.R. part 56.

†
http://www.lakecountyca.gov/Government/PressReleases/TapWater091621.htm

§
http://www.lakecountyca.gov/Government/PressReleases/11172021.htm

¶
https://www.epa.gov/nutrientpollution/climate-change-and-harmful-algal-blooms
